# Tung tree stearoyl‐acyl carrier protein Δ9 desaturase improves oil content and cold resistance of *Arabidopsis* and *Saccharomyces cerevisiae*

**DOI:** 10.3389/fpls.2023.1144853

**Published:** 2023-03-07

**Authors:** Junjie Chen, Jing Gao, Lingling Zhang, Lin Zhang

**Affiliations:** ^1^ Key Laboratory of Cultivation and Protection for Non-Wood Forest Trees, Ministry of Education, Central South University of Forestry and Technology, Changsha, China; ^2^ CAS Key Laboratory of Plant Germplasm Enhancement and Specialty Agriculture, Wuhan Botanical Garden, The Innovative Academy of Seed Design, Chinese Academy of Sciences, Wuhan, China; ^3^ Center of Economic Botany, Core Botanical Gardens, Chinese Academy of Sciences, Wuhan, China; ^4^ University of Chinese Academy of Sciences, Beijing, China

**Keywords:** tung tree (*Vernicia fordii*), stearoyl-acyl carrier prote in Δ9 desaturase (SAD), oleic Acid, the α-eleostearic acid, oil accumulation

## Abstract

The seed oil of tung tree is rich in a-eleostearic acid (ESA), which endows tung oil with the characteristic of an excellently dry oil. The stearoyl-acyl carrier protein δ9 desaturase (SAD) is a rate-limiting enzyme that converts the stearic acid to the oleic acid, the substrate for the production of the α-ESA. However, the function of the two predicted *VfSAD1* and *VfSAD2* genes in the tung tree has not been determined. In this study, quantitative real-time PCR (qRT-PCR) analysis showed that *VfSAD1* and *VfSAD2* were expressed in multiple organs of tung tree but were highly expressed in the seed during the oil rapid accumulation period. Heterologous expression of *VfSAD1* and *VfSAD2* could promote the production of oleic acid and its derivatives in Arabidopsis thaliana and yeast BY4741, indicating that *VfSAD1* and *VfSAD2* possess the stearoyl-ACP desaturases function. Furthermore, both *VfSAD1* and *VfSAD2* could significantly improve seed oil accumulation in Arabidopsis. VfSAD1 could also significantly promote the oil accumulation in the yeast BY4741 strain. In addition, overexpression of *VfSAD1* and *VfSAD2* enhanced the tolerance of yeast and Arabidopsis seedlings to low temperature stress. This study indicates that the two *VfSAD* genes play a vital role in the process of oil accumulation and fatty acid biosynthesis in the tung tree seed, and both of them could be used for molecular breeding in tung tree and other oil crops.

## 1 Introduction

Tung tree (*Vernicia fordii*) is an oil-bearing woody plant of Euphorbiaceae. The oil extracted from the fruits of the tung tree, also called tung oil, is a superior drying oil, which is widely used in multiple aspects of the industry. Due to its economic value, tung tree has been cultivated in China for over 1,000 years in history. The plant’s oil quality is largely determined by the composition of fatty acid. The α-eleostearic acid (α-ESA, 18:3^Δ9cis, 11trans, 13trans^) is an unusual fatty acid, which contains three conjugated unsaturated double bonds (Dyer et al., 2002). It accounts for more than 80% of fatty acid components in the tung oil and thus endows the tung oil with active chemical activity.

Triacylglycerol is the main storage form of plant oil and the most important energy supplier for plant growth and development. Lots of efforts have been made to detect the complex molecular mechanism for seed oil accumulation (Shockey et al., 2016; Zhang et al., 2021; Nam et al., 2022; Yang et al., 2022). At this background, tung tree genome was published (Cui et al., 2018; Zhang et al., 2019), which benefited the deep investigation of tung oil accumulation at the molecular level. Tung oil quickly accumulated from early August to late September (Galli et al., 2014). Meanwhile, transcriptome analysis revealed that a large body of the genes involved in oil biosynthesis and accumulation were upregulated and kept a high expression pattern during the oil rapid accumulation period and then was sharply downregulated at the late period of oil accumulation (Cui et al., 2018; Zhang et al., 2019). The tung tree genotype with the higher seed oil content, to a large degree, was due to the extended phase of the high expression level of those global genes involved in oil biosynthesis and accumulation (Zhang et al., 2018). In addition, efforts were also made to detect the critical transcript factors that were involved in tung oil accumulation during the oil accumulation period (Zhang et al., 2018; Zhang et al., 2021). The biosynthetic pathway of tung oil is shown in **Supplementary Figure S1**. In the seed of tung tree, the oleic acid synthesized in the plastid is transported to the ER, where the oleic acid is further modified by the FAD2 and FADX enzymes to form the α-ESA (Dyer et al., 2002). The fatty acid molecules in the ER were then assembled into TAG mainly by two pathways, the Kenney pathway and the PC-derived DAG pathway (Li-Beisson et al., 2013). Up to now, little is known about the molecular mechanism responsible for the high percentage of the α-ESA in tung oil (van Erp et al., 2015; Yurchenko et al., 2017). Thus, there is an urgent need to investigate the function of the components in the tung oil biosynthetic complex.

Stearoyl-acyl carrier protein Δ9 desaturase (SAD) is an important rate-limiting enzyme that controls the desaturation of stearic acid to the oleic acid during the process of fatty acid *de novo* synthesis in the plastid. Up to now, the desaturases of SAD derived from various plants have been investigated by heterologous expression (Li et al., 2020). The oleic acid produced by the SAD could be assembled into different lipid classes. Thus, the *SAD* genes actually participated in multiple biological processes during the growth and development of plants. In *Arabidopsis*, it was proven that the SAD members participated in the embryo development, elaboration of the embryonic cuticle, and storage of lipid production during the seed maturation phase (Kazaz et al., 2020). During the oil rapid accumulation period, two predicted *SAD* genes were highly expressed in the seeds of tung tree. However, the function of the *SAD* genes from tung tree during seed oil accumulation has not been studied. In this study, the *VfSAD*1 and *VfSAD2* are proven to be the stearoyl-ACP Desaturases. It could improve the lipid accumulation in the seed of *Arabidopsis* and enhance the tolerance of *Arabidopsis* seedlings to low-temperature stress. Similar results were obtained by the heterologous expression of the two *VfSAD* genes in the yeast BY4741 strains. This study indicated that the two *SAD* genes should play a crucial role in the seed oil accumulation of tung tree.

## 2 Materials and methods

### 2.1 Plant materials and methods

Tung tree fruits were collected from tung tree germplasm nursery established in Wuhan, Hubei, China, in 2021. Lipid accumulation in tung tree seeds began in July, accumulated rapidly in August, and stabilized in September. We aim to focus on the conversion of saturated fatty acids to unsaturated fatty acids. Based on this process, we selected five representative lipid accumulation periods. Tung fruits were collected at five stages of seed development (including 26 Jul, 2 August, 16 August, 30 August, and 13 September). Three fruits were taken each time, each fruit as one biological repeat. The endosperm tissue was ground into powder in liquid nitrogen for oil content measurement. *Arabidopsis thaliana* was grown in the greenhouse at 26°C and 60% relative humidity with a 14-h light/10-h dark–light cycle.

### 2.2 RNA isolation, inversion, and real-time quantitative PCR

Total RNA was extracted from the seeds of tung fruit an RNA extraction kit (BioTeck Corporation, China). RNA purity and RNA concentrations were detected by a nanophotometer (IMPLEN, CA, USA). According to the manufacturer’s instructions, the first-strand cDNA was reverse-transcribed using PrimeScript™ RT Kit (Cat. RR047A, TaKaRa, Japan). The reaction solution contained SYBR^®^ PremixExTaq™ II (Tli RNaseH Plus) (TaKaRa, Japan). The gene-specific primers used for qPCR are shown in **Supplementary Table S1**. Applied Biosystems™ QuantStudio™ 6 Flex Real-Time PCR System was used in the experiment, with a total of three replicates. Reaction conditions were 95°C for 30 s, 95°C for 10 s, 60°C for 30 s, and 40 cycles. After the final cycle, the melting curve was analyzed, rising from 60°C to 95°C with an increase of 0.3°C/5s. The 2^−ΔΔCT^ method was used to analyze the fluorescence quantitative PCR data (Livak and Schmittgen, 2001).

### 2.3 Sequence analysis of VfSAD1 and VfSAD2 proteins from *Vernicia fordii*

The physical and chemical properties were analyzed by using ProtParam online tool (http://web.expasy.org/protparam/). Signal peptide prediction SignalP4.0 server (http://www.cbs.dtu.dk/services/Sig-nalP/) was to predict VfSAD1 and VfSAD2 amino acid sequence of the splice site if there was a potential signal peptide and its place. The online software protscale was used to analyze the hydrophobicity of VfSAD1 and VfSAD2. Tmpred website was used to predict the transmembrane domain of VfSAD1 and VfSAD2 proteins and at the same time submit the amino acid sequence to online analysis software for protein secondary structure prediction (https://www.predictprotein.org/). The tertiary structure of proteins was modeled by SWISS-MODEL prediction and modeling (https://swissmodel.expasy.Org). Multiple protein sequences were aligned with Geneious software, and the alignment results were compared with MAGE7.0 software to construct a phylogenetic tree using the neighbor-joining method.

### 2.4 Subcellular localization

The *Agrobacterium* infection method was applied to infuse *Agrobacterium* containing *35S: VfSAD1*-GFP, *35S: VfSAD1*-GFP, and *Agrobacterium* containing plasmid location Marker 217: mCherry into the lower epidermal cells of wild-type tobacco leaves, respectively. After dark culture for 24 h at room temperature and 48 h under normal light, the co-localization of proteins encoded by *VfSAD1* and *VfSAD2* genes and chloroplast Marker 217: mCherry was observed under laser confocal three-dimensional scanner (TCS SP8).

### 2.5 Measurement of oil content and fatty acid components

The oil content of tung tree seeds was measured using gas chromatography (GC, Agilent 7820A) according to the method of Zhang et al. (2018). The measurement of oil content and fatty acid components in the mature seed of *Arabidopsis* was referred to Li-Beisson et al. (2013). The total oil content of *Saccharomyces cerevisiae* BY4741 in 20 ml of SD-Trp liquid medium was measured. Analyses of fatty acid composition and oil content in BY4741 were referred to Wu et al. (2018). The fatty acid percentage is calculated according to the percentage of fatty acid peak area. The oil content is calculated by the following formula:


$$\frac{\left(\frac{(\text{Total peak area}-{\text{C}}_{17}:\text{o}(\text{internal standard})\text{area})=\text{Peak area of oil}}{C_{17}:\text{o}(\text{internal standard})\text{peak area}}\right)\times \text{The internal standard dosage}}{\text{Sample seed number}(\text{or sample weight})}$$

Note: The internal standard dosage in this study was 20 mg for *Arabidopsis* seeds, 50–80 mg for tung tree seeds (select specific dosage according to different lipid content), and 50 mg for yeast strains.

The error bar represents the standard deviation ( ± SD) for setting three biological replicates. For example, statistical analysis of data such as oil content and 1,000-grain weight of seeds was conducted by using *t*-test (* means *p*< 0.05; ** means *p*< 0.01). A comparison between groups was conducted by one-way ANOVA test (FA component), and the different letters above the bar chart indicated significant differences (p< 0.05).

### 2.6 Low-temperature stress treatments of transgenic yeasts

*VfSAD1/2* was used to construct the pESC-Trp expression vector in yeast experiments; after extracting the plasmid, the yeast strain BY4741 was transformed to verify the function of SAD dehydrogenase.

In this experiment, three to five repeated bacterial liquids and colonies were selected, screened by glucose medium, and cleaned with sterile water, and underwent a galactose-induced expression program, at 17°C and 30°C, and the OD600 value of the colony was focused on the performance after 12 h. The solid medium culture was a unified dilution concentration multiple (10^0^, 10^−1^, 10^−2^, 10^−3^, 10^−4^, 10^−5^, and 10^−6^).

### 27 Low-temperature stress treatments of transgenic *Arabidopsis*

In this study, the Columbia ecotype of *Arabidopsis thaliana* was used as control. According to the method of Huang et al. (2022), transgenic *Arabidopsis* seeds were sterilized, and 25 seeds (5×5 rows) were placed on MS solid medium (MS 4.43 g/L+ sucrose 10 g/L + agar 8 g/L) using toothpicks. To achieve consistent germination and growth of *Arabidopsis* seeds, the seeds were placed in 4°C darkness for 2 days for vernalization (vernalization treatment). After fully absorbing water, all seeds were placed in a plant room (24°C under a 16-h light/8-h dark cycle with a photon flux of 240 µmol/m/s) for a week. *Arabidopsis thaliana* materials were placed in an incubator at −8°C for 1–3 h, the phenotype was observed, growth was recovered at 24°C for 3 days, and then the survival rate was calculated.

## 3 Results

### 3.1 Molecular identification and sequence analysis of *VfSAD1* and *VfSAD2* genes from tung tree seed

**Figure 1:**
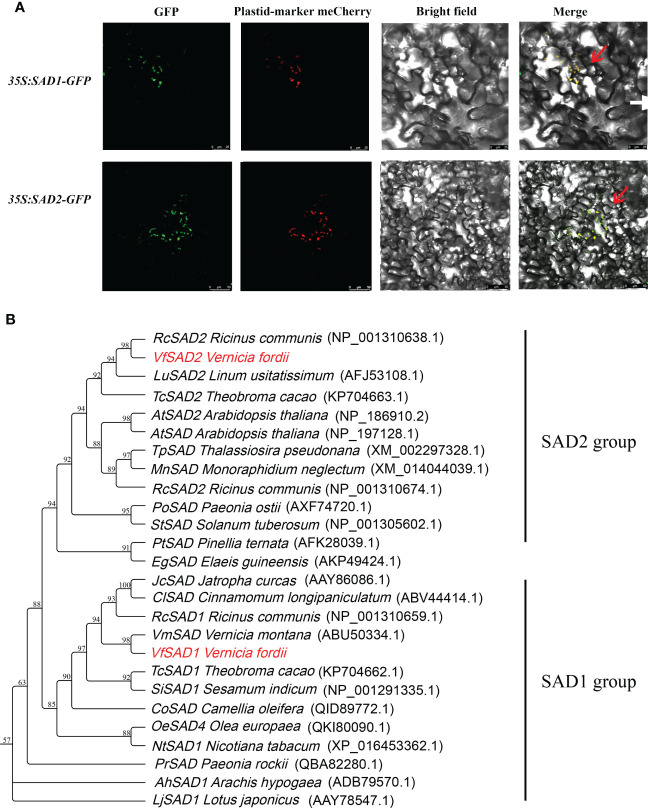
The characteristic analysis of VfSAD1 and VfSAD2 protein. **(A)** Subcellular localization of VfSAD1 and VfSAD2 proteins. **(B)** Phylogenetic tree analysis of SAD proteins from tung tree and other species.

We identified three SAD members using the genome data, but only two were upregulated in the lipid synthesis pathway. These two genes were predicted as stearoyl-acyl carrier protein Δ9 desaturase (SAD). Based on tung tree genomic data, the sequences of the two genes, named *VfSAD1* and *VfSAD2*, were obtained. The open reading frame length of *VfSAD1* was 1,191 bp, encoding 397 amino acids, and the open reading frame length of *VfSAD2* was 1,176 bp, encoding 386 amino acids (**Supplementary Table S2**). Subcellular localization analysis revealed that *VfSAD1* and *VfSAD2* were both located on chloroplasts (**Figure 1A**). Using ProtParam online tool analysis, the molecular formula of VfSAD1 is C_3601_H_6013_N_1191_O_1507_S_247_, and its molecular weight is approximately 45.54 kDa. The molecular formula of VfSAD2 is C_3541_H_5908_N_1176_O_1477_S_255_, and its molecular weight is approximately 43.82 kDa. Other parameters of physicochemical properties of VfSAD1 and VfSAD2 proteins are listed in **Supplementary Table S2**. The protein secondary structure of VfSAD1 and VfSAD2 was analyzed using online software (https://www.predictprotein.org/). The results showed that VfSAD1 and VfSAD2 proteins had a similar secondary structure (**Supplementary Figure S2A**). In addition, multiple sequence alignment showed that VfSAD1 and VfSAD2 proteins are highly conserved among plant SAD members (**Supplementary Figure S3A**). Furthermore, the phylogenetic tree of *VfSADs* was constructed using the adjacency method through MAGE5.0 software. As shown in **Figure 1B**, *VfSAD1* was closely related to *Vernicia montana* Lour, and *VfSAD2* was closely related to *Ricinus communis*.

### 3.2 The mRNA levels of *VfSAD1* and *VfSAD2* were largely upregulated in the seed during the oil accumulation period

**Figure 2:**
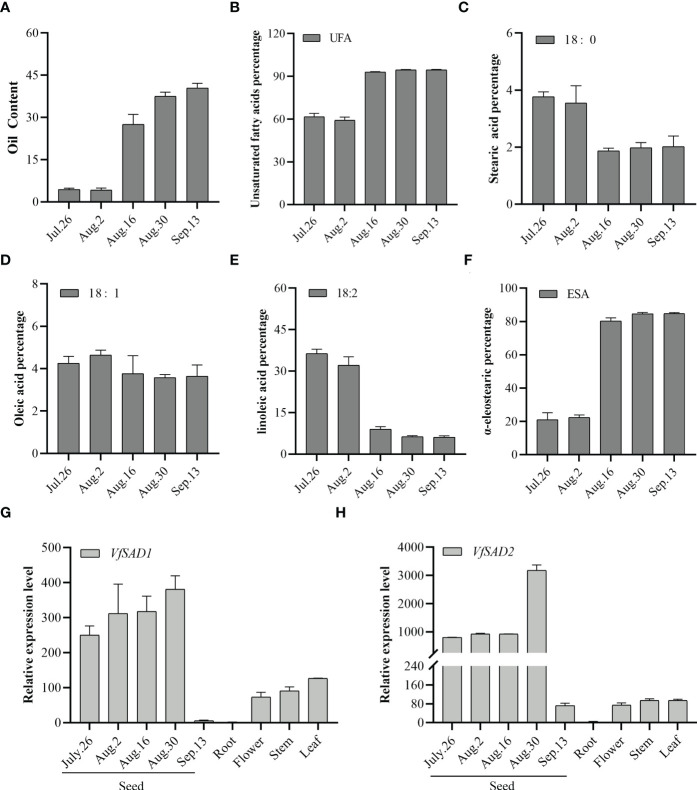
Oil content in the developing seed and the expression level of the two *SAD* genes in the multiple tissues of tung tree. **(A)** Oil content in the seeds from multiples development stages; **(B–F)** percentage of unsaturated fatty acid **(B)**, stearic acid **(C)**, oleic acid **(D)**, linoleic acid **(E)**, and α-eleostearic **(F)** in the seeds from multiples development stages; **(G, H)** the relative expression level of *VfSAD1* **(G)** and *VfSAD2* **(H)** in the different tissues of tung tree. The error bar represents the standard deviation (± SD) for setting three biological replicates.

The expression of *VfSAD1/2* gene in different tissue parts and seed kernels at five different developmental stages was analyzed by quantitative real-time PCR (qRT-PCR). The accumulation of oil in the seeds of *Aleurites* mainly comes from the endosperm gradually expanded at the later stage of kernel maturation. As shown in **Figures 2G and H**, the *VfSAD1/2* gene was expressed in the kernel, flower, stem, and leaf of tung tree, and the expression level in the kernel was higher than that in other tissues. In this study, seed kernel expression was detected at the five developmental stages (**Figure 2**). The expression level of *VfSAD1* and *VfSAD2* genes began to increase gradually on 26 July and increased rapidly to the highest level on 30 August (at the stage of lipid synthesis). After reaching the peak, the expression level of *VfSAD1* and *VfSAD2* genes decreased rapidly to the lowest level on 3 September (at the end of lipid accumulation). Based on the expression patterns of *VfSAD* genes, it was concluded that *VfSAD1* and *VfSAD2* may be involved in lipid synthesis and storage during the development of tung tree seeds.

By analyzing the samples from the five periods, it was found that before 2 August, the lipid content was low (**Figure 2A**), and the unsaturated fatty acids accounted for approximately 60% of the total fatty acids (**Figure 2B**). But since 16 August, lipids have begun to accumulate in large quantities, with unsaturated fatty acids increased sharply to 90%. The unsaturated fatty acids in IL tung oil mainly include oleic acid, linoleic acid, and α-ESA. As shown in **Figures 2C–E**, oleic acid content was stable. Linoleic acid content decreased sharply since 16 August, but alpha-ESA content increased dramatically and accounted for more than 80% of the total fatty acids (**Figure 2F**). The results showed that stearic acid could be efficiently converted to oleic acid, and oleic acid could be efficiently converted to α-ESA. Oil accumulation and fatty acid conversion tended to stop at a late stage of seed development. Similarly, according to **Figures 2G, H**, *VfSAD1/2* expression decreased sharply. These results suggested that the *VfSAD1* and *VfSAD2* genes are mainly involved in regulating the transformation of saturated fatty acid stearic acid in tung tree seeds, thereby increasing the amount of biosynthesis of unsaturated fatty acid–oleic acid and other products.

### 3.3 Overexpression of *VfSAD1* and *VfSAD2* could change the composition of fatty acids

**Figure 3:**
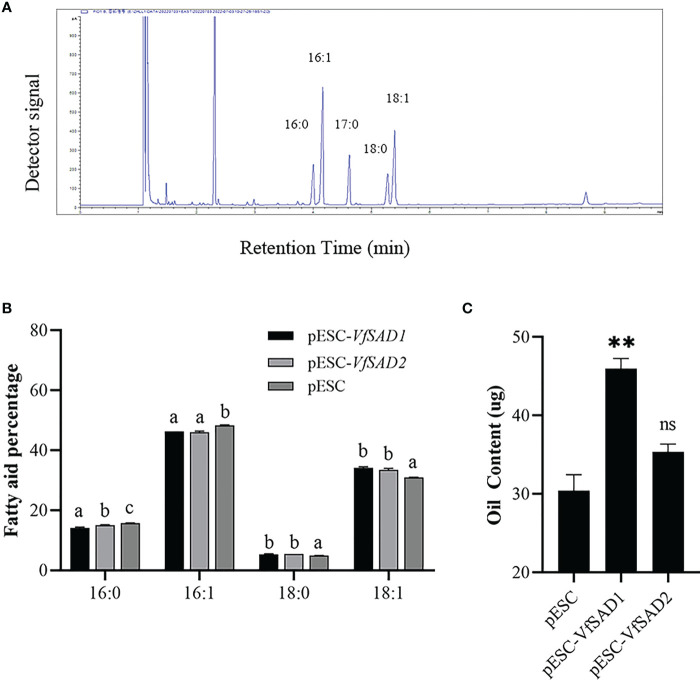
The *VfSAD1/2* improves oil content and alters the percentage of the fatty acid component in the *S. cerevisiae* BY4741. **(A)** GC-FID analysis of fatty acid methyl esters derived from neutral lipids isolated from *S. cerevisiae* BY4741. **(B)** The percentage of fatty acid components in the wild-type BY4741 containing the empty plastid and transgenic BY4741 with the overexpression of *VfSAD1* or *VfSAD2*. **(C)** Oil content in the wild-type BY4741 and transgenic BY4741 containing *VfSAD1* or *VfSAD2*. The error bar represents the standard deviation (± SD) for setting three biological replicates. Significant difference was detected by *t*-test (* indicates *p*< 0.05; ** indicates *p*< 0.01). A comparison between groups was conducted by one-way ANOVA test (FA component), and the different letters above the bar chart indicate significant differences (p< 0.05).

The *VfSAD1* and *VfSAD2* were investigated in the *S. cerevisiae* BY4741 to determine the biochemical function. Thus, *VfSAD1* and *VfSAD2* were transferred into BY4741, respectively. The fatty acid in the oil of BY4741 contained palmitic acid, palmitoleic acid, stearic acid, and oleic acid (**Figure 3A**). The fatty acid profile demonstrated that *VfSAD1* and *VfSAD2* altered the percentage of the fatty acid component in the oil of BY4741. The content of oleic acid changed the most, increasing by approximately 10% (**Figure 3B**) in the BY4741 strain containing *VfSAD1* or *VfSAD2* gene. In addition, the stearic acid was significantly increased, and the palmitic acid and palmitoleic acid were significantly decreased in the BY4741 containing *VfSAD1* or *VfSAD2* gene in comparison with that containing the empty plasmid. The result reflected that *VfSAD1* and *VfSAD2* promoted the production of oleic acid and thus led to the percentage alteration of the palmitic acid, palmitoleic acid, and stearic acid in the lipid of BY4741.

**Figure 4:**
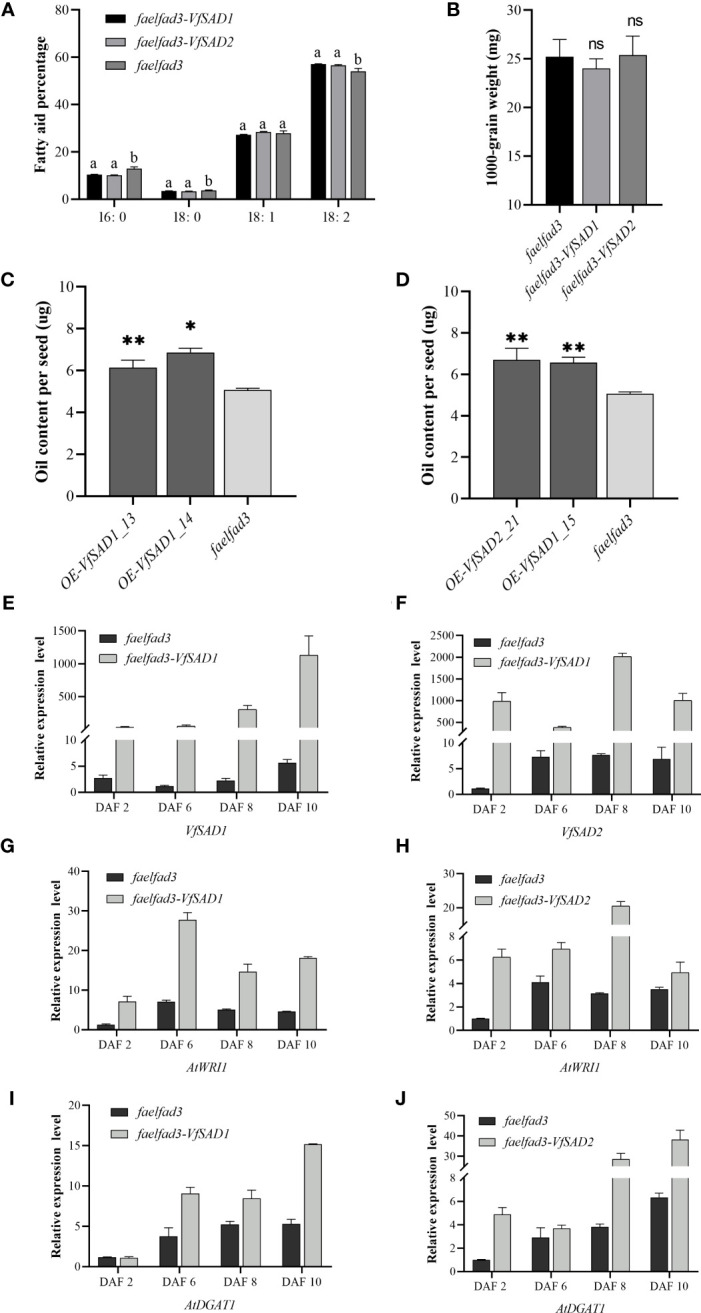
The overexpression of *VfSAD1/2* altered the percentage of fatty acid components and oil content in the seeds of *Arabidopsis*. **(A)** Fatty acid composition in seeds of *SAD1-OE* line, *SAD2-OE* line, and *fad3fae1* line. **(B)** The 1,000-grain weight in seeds of *SAD1-OE* line, *SAD2-OE* line, and *fad3fae1* line. **(C)** Oil content in seeds of *SAD1*-OE lines and *fad3fae1* line. **(D)** Oil content in seeds of *SAD2*-OE lines and *fad3fae1* line. **(E)** The expression pattern of *VfSAD1* in the *SAD1-OE* line and *fad3fae1* line. **(F)** The expression pattern of *VfSAD2* in the *SAD2-OE* line and *fad3fae1* line. **(G)** The expression pattern of *AtWRI1* in the *SAD1-OE* line and *fad3fae1* line. **(H)** The expression pattern of *AtWRI1* in the *SAD2-OE* line and *fad3fae1* line. **(I)** The expression pattern of *AtDGAT1* in the *SAD1-OE* line and *fad3fae1* line. **(J)** The expression pattern of *AtDGAT1* in the *SAD2-OE* line and *fad3fae1* line. The error bar represents the standard deviation (± SD) for setting three biological replicates. Significant difference was detected by *t*-test (* indicates *p*< 0.05; ** indicates *p*< 0.01). A comparison between groups was conducted by one-way ANOVA test (FA component), and the different letters above the bar chart indicate significant differences (*p*< 0.05).

To further confirm the biochemical function of *VfSAD1* and *VfSAD2*, overexpression was performed in the *fad3fae1* double mutant of the accession Landsberg (Ler) of *A. thaliana*. The fatty acid components were analyzed in the seed of *VfSAD1/2*-overexpression (*VfSAD1/2*-OE) lines and the *fad3fae1* line. The oleic acid and linoleic acid accounted for the largest proportion in the *fad3fae1* line. Compared with the *fad3fae1* line, oleic acid content was not obviously changed in the seeds of *VfSAD1/2*-OE lines, but its derivation, linoleic acid, was significantly increased. The palmitic acid and stearic acid content were also significantly decreased in the seeds of *VfSAD1/2*-OE lines (**Figure 4A**). The result reflected that *VfSAD1* and *VfSAD2* promoted the production of oleic acid that was further converted to linoleic acid in the seed of *VfSAD1/2*-OE lines. Thus, the results above revealed that the two *SADs* of tung tree were the stearoyl-ACP desaturases.

### 3.4 Overexpression of *VfSAD1* and *VfSAD2* could improve oil accumulation

To detect the influence of the overexpression of *VfSAD1* and *VfSAD2* on oil accumulation, the oil content in the yeast and seed of *Arabidopsis* was measured. In the yeast, the oil content was significantly increased in the BY4741containing *VfSAD1* gene, with an increase of 30%–34% but was not in the BY4741 strain containing *VfSAD2* gene (**Figure 3C**). In *Arabidopsis*, the seed oil content was significantly improved in the *VfSAD1*-OE and *VfSAD2*-OE lines than that in the *fad3fae1* line (**Figures 4C, D**). Compared with fad3fae1, the oil content was increased by 21.1% and 35.3% in the three homozygous VfSAD1-OE lines, respectively (**Figure 4C**). The oil content was increased by 29.4% and 32.2% in the three homozygous VfSAD2-OE lines, respectively (**Figure 4D**). In addition, it was found that seed weight was not obviously altered in the *VfSAD1/2*-OE lines in comparison to the *fad3fae1* line (**Figure 4B**). The results further indicated that overexpression of *VfSAD1* and *VfSAD2* promoted the accumulation of oil.

### 3.5 The expression of *AtWRI1* and *AtDGAT1* was upregulated in the *VfSAD1/2-OE* lines

*WRI1* is a master transcriptome factor that directly regulates multiple genes involved in the fatty acid *de novo* biosynthesis (To et al., 2012). *DGAT1* is one of the diacylglycerol acyltransferases, which has been proven to play a vital role in seed oil accumulation in *Arabidopsis* (Zou et al., 1999). To detect the molecular mechanism for the oil content improvement in the *VfSAD1/2-OE* lines, the gene expression patterns of *AtWRI1* and *AtDGAT1* were detected in the developing silique of *Arabidopsis*. The developing siliques were sampled at 2, 6, 8, and 10 days after flowering (DAF). The *VfSAD1*/*2* showed high expression in the siliques of *VfSAD1/2-OE* lines at the four development periods (**Figures 4E, F**). As shown in **Figures 4G–J**, the expression levels of the *AtWRI1* and *AtDGAT1* in the siliques of *VfSAD1/2-OE* lines were all significantly upregulated in at least two of the four development periods in comparison to that in the *fad3fae1* line. The results indicated that the improved oil content in the *VfSAD1/2-OE* lines should be due to the upregulation of the genes involved in the oil biosynthesis pathway.

### 3.6 *VfSAD1* and *VfSAD2* could enhance the tolerance of yeast and *Arabidopsis* seedlings to the low temperature stress

**Figure 5:**
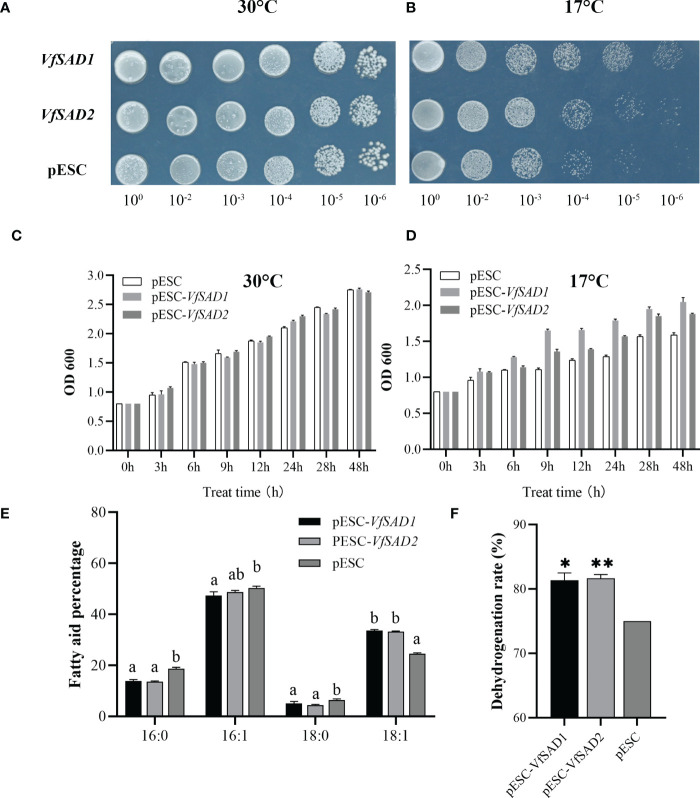
VfSAD1 and VfSAD2 enhance the tolerance of yeast to low temperature stress. **(A, B)** Yeast colonies at 30°C **(A)** and 17°C **(B)**; **(C, D)** OD values of yeast liquid cultures treated at 30°C **(C)** and 17°C **(D)**; **(E)** fatty acid fractions in the BY4741 treated at 17°C; **(F)** conversion of unsaturated fatty acids in the BY4741 treated at 17°C. The error bar represents the standard deviation (± SD) for setting three biological replicates. Significant difference was detected by *t*-test (* indicates *p*< 0.05; ** indicates *p*< 0.01). A comparison between groups was conducted by one-way ANOVA test (FA component), and the different letters above the bar chart indicate significant differences (*p*< 0.05).

The increase in unsaturated FAs content in the cell membrane enhanced the membrane lipid fluidity and thus could increase the tolerance of the cell to the low temperature (Xiao et al., 2022). The results above showed that the overexpression of *VfSAD1/2* promoted the production of oleic acid and/or its derivation in the BY4741 and *Arabidopsis*. The optimal growth temperature for yeast is 28°C–30°C. In order to detect the tolerance of transgenic BY4741 strain with the overexpression of the *VfSAD1* or *VfSAD2* gene to the low temperature, two temperature values, 17°C and 30°C, were selected for the experiment. At 30°C, there was no difference in growth performance between wild-type BY4741 and transgenic BY4741 at six diluted concentrations (**Figure 5A**). However, under the condition of 17°C, the growth activity of transgenic BY4741 was significantly better than that of the wild type (**Figure 5B**).

Furthermore, the time-course optical density (OD) value of the two kinds of yeast was measured. The initial OD values of the two types of yeast were adjusted to 0.8. At 30°C, there was no obvious difference in OD value between the two types of yeasts (**Figure 5C**) at the different growth stages. The OD values of transgenic BY4741 containing *VfSAD1* or *VfSAD2* were obviously lower at 17°C than that at 30°C since growth for 6 h. However, the OD values of transgenic BY4741 containing *VfSAD1* or *VfSAD2* were significantly higher than that of the wild-type BY4741 at each sampling time at the 17°C (**Figure 5D**). The results reflected that the low temperature decreased the growth rate of yeast, but the overexpression of *VfSAD1/2* could partly recover the growth rate of yeast. The oleic acid percentage in the transgenic BY4741 containing *VfSAD1* or *VfSAD2* was increased by 10% than that in the wild-type BY4741 at 30°C (**Figure 3A**). Here, at 17°C, the oleic acid was further increased by 13.7% in the transgenic BY4741 containing *VfSAD1* or *VfSAD2* than that in the wild-type BY4741, and other three fatty acids, the palmitic acid, palmitoleic acid, and saturated acid, were all significantly decreased than that in the wild-type BY4741 (**Figures 5E, F**). This result reflected that more oleic acid molecules were produced in the transgenic BY4741 under low temperature.

**Figure 6:**
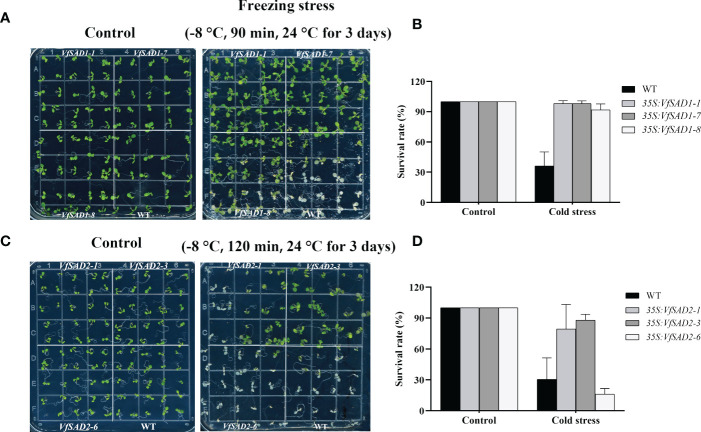
*VfSAD1* and *VfSAD2* enhance the tolerance of *Arabidopsis* seedlings to low temperature stress. **(A, B)** Phenotypes **(A)** and survival rates **(B)** of the *VfSAD1*-overexpressing and WT *Arabidopsis plants* under normal and freezing treatments (−8°C for 90 min); **(C, D)** phenotypes **(C)** and survival rates **(D)** of the *VfSAD2*-overexpressing and WT *Arabidopsis plants* under normal and freezing treatments (−8°C for 120 min). The error bar represents the standard deviation (± SD) for setting three biological replicates.

To understand the biological function of *VfSAD1/2* in regulating cold tolerance, three *VfSAD1/2*-overexpressing (OE) *Arabidopsis* lines with relatively high expression were selected for assessing cold tolerance. No conspicuous difference in phenotype was observed between transgenic *Arabidopsis* plants and WT under normal conditions (**Figures 6A, B**). After the treatment at −8°C for 90 min, the wild *Arabidopsis* leaves showed severe wilting, whereas the *VfSAD1-OE Arabidopsis* leaves exhibited slight wilting. In the subsequent 3 days of recovery culture, the wild-type *Arabidopsis* plants almost died (**Figures 6A, C**), but the *VfSAD1-OE Arabidopsis* grew larger. The average survival rate of the three strains of *VfSAD1* was 98%, 98.1%, and 91.75%, respectively, and the average survival rate of WT was 36.1% (**Figure 6B**). Little difference was observed between transgenic *Arabidopsis* plants and WT after the treatment at −8°C for 90 min. Thus, the *VfSAD2-OE Arabidopsis* was treated at −8°C for 2 h and then recovered at 24°C for 3 days. The phenotype of the *VfSAD2-OE Arabidopsis* to the low temperature was similar to that of the *VfSAD1-OE Arabidopsis*. The average survival rate of the three transgenic plant lines was 79.4%, 88%, and 16%, respectively, and the average survival rate of the WT was 30.5% (**Figure 6D**). Therefore, tolerance to low temperature was significantly improved in the 7-day-old plantlet of *A.* thaliana with overexpression of *VfSAD1* or *VfSAD2* gene (**Figure 6**). The results above indicated that under low temperature conditions, the overexpression of the *VfSAD1* or *VfSAD2* could improve the tolerance of the cell to the low temperature stress.

## 4 Discussion

There are usually one to four members of the *SAD* gene for each plant species according to other existing studies. In this study, we identified three SAD members using the genome data, but only two were upregulated in the lipid synthesis pathway, so we annotated and downloaded the sequences of VfSAD1 and VfSAD2 using genomic data to focus on their functional characterization (Zhang et al., 2019). Phylogenetic analysis showed that *SAD1* and *SAD2* genes were clustered into different groups, suggesting genetic differentiation between *SAD1* and *SAD2*, which is consistent with the report of Li et al. (2022). Despite the genetic differentiation between the *SAD1* and *SAD2*, we observed that they displayed similar functions in fatty acid biosynthesis. Overexpression of *VfSAD1* or *VfSAD2* can improve oleic acid content and cold resistance in transgenic yeast and *Arabidopsis*. During low-temperature acclimation of higher plants, polyunsaturated FAs have been found to increase in many plant species (De Palma et al., 2008; Wang et al., 2013). The researchers found that the *SAD* genes were upregulated and the content of unsaturated fatty acid increased in response to the low temperature (Ding et al., 2016; Hernández et al., 2019; Zhou et al., 2019).

The function of a *SAD* gene is usually considered to catalyze the formation of oleic acid with stearic acid as the substrate. However, we found that overexpression of both *VfSADs* could increase oil content in addition to oleic acid content in transgenic *Arabidopsis*. Actually, Kazaz et al. (2020) reported that decreased *SAD* gene expression leads to the decreased oil content in *Arabidopsis* seeds. Association analysis further confirmed that *GhSAD4* plays an important role in cottonseed oil composition (Shang et al., 2017), which supports our finding that *SAD* is also involved in oil accumulation. Interestingly, we also found that *AtWRI1* and *AtDGAT1* were upregulated in *VfSAD* transgenic *Arabidopsis*. *AtWRI1* and *AtDGAT1* are two key genes involved in oil biosynthesis. A *WRI1* transcription factor is considered as a “master regulator” that controls the transcription of almost all key enzymes converting sucrose to FA (Liu et al., 2019). For instance, seed oil decreased significantly in *Arabidopsis* wri1 mutants (An et al., 2017), and overexpression of *WRI1* increased seed oil content in several plant species (Snell et al., 2019; Fei et al., 2020; Sanchez et al., 2022). The *DGAT* protein family catalyzes the final step in triacylglycerol (TAG) biosynthesis, which is considered to be the rate-limiting step in TAG accumulation (Shockey et al., 2006; Cao et al., 2013). According to the study of Zhang et al. (2019), *DGAT2* was considered to be the most important *DGAT* gene for TAG biosynthesis in tung tree seeds. Since the expression of *SAD* causes the first step of the saturated fatty acid dehydrogenation reaction, we speculate that this reaction may accelerate the whole oil biosynthesis pathway, and then, the expression level of *WRI1* and *DGAT1* are upregulated.

In addition, we noted that *VfSAD1* showed a higher expression level than *VfSAD2* in developing tung tree seeds, suggesting that *VfSAD1* may play a more important role than *VfSAD2* in oil and fatty acid biosynthesis. However, overexpression of the two genes in *Arabidopsis* generated opposite results, namely, the transgenic *Arabidopsis* with *SAD2* produced higher oil content and oleic acid content than the *VfSAD1* carrying *Arabidopsis*. This inconsistent finding may be due to two reasons. First, expression patterns imply a biological function for genes, while only higher expression does not represent stronger biological function, since the function of genes is also related to the posttranslational modification of proteins. Second, it is generally believed that the integration site of transgenic gene in the host chromosome is random. Exogenous DNA can be inserted into any chromosome of the plant genome, or any site of a chromosome, or there is no fixed insertion site, but it often has priority insertion characteristics for the transcription active region. Thus, the phenotype of transgenic lines is not only related to the function of exogenous genes but also to the insertion sites in the host genome. To compare the significance of the two *VfSADs* in oil and fatty acid biosynthesis, in fact, gene knockout dependent on genetic transformation system is the most efficient method. This transformation system, however, has not yet been resolved. Based on our present results, both *VfSADs* seem to play a key role with almost identical efficiency in oil and fatty acid biosynthesis. In our previous study, we have developed a method of *Agrobacterium* rhizogenes-mediated transgenic hairy root induction from the stem of tung tree (Jia et al., 2022), which laid an important foundation for the construction of a genetic transformation system. The two *VfSADs* could be used for tung tree genetic improvements, e.g., increase seed oil content and oleic acid proportion or improve cold resistance once the genetic transformation system of tung tree is successfully established in the future.

## 5 Conclusion

The two predicted *VfSAD1* and *VfSAD2* were highly expressed in the seed of tung tree during the oil rapid accumulation period. The function of two predicted *SAD* genes was investigated in *Arabidopsis* and yeast. The results showed that the *VfSAD*1 and *VfSAD2* are the stearoyl-ACP desaturases. It could improve the lipid accumulation in *Arabidopsi*s seed and yeast and enhance the tolerance of yeast and *Arabidopsis* seedlings to the low temperature stress. This study indicated that the two *SAD* genes should play a vital role in the process of oil accumulation in the seed of tung tree.

## Data availability statement

The datasets presented in this study can be found in online repositories. The names of the repository/repositories and accession number(s) can be found in the article/**Supplementary Material**.

## Author contributions

LZ and LLZ contributed to conception and design of the study. LZ contributed to supervision and writing—reviewing and editing. JC organized the database. JG performed the statistical analysis by using software. JC wrote the first draft of the manuscript and performed validation and visualization. LZ, LLZ, and JC wrote sections of the manuscript. All authors contributed to manuscript revision, read, and approved the submitted version.
