# Supplementary material for: Tung tree stearoyl‐acyl carrier protein Δ9 desaturase improves oil content and cold resistance of Arabidopsis and Saccharomyces cerevisiae
Source: Front Plant Sci. 2023 Mar 7;14:1144853. doi: 10.3389/fpls.2023.1144853 (PMC10028071; doi:10.3389/fpls.2023.1144853)
Supplement: Supplementary file 1 [file DataSheet_1.docx]

**Appendix A. Supplementary material**

**Table S1. Primers used for quantitative analysis.**

| Gene Name | Amplicon | Forward primer (5' to 3') | Product length |
| --- | --- | --- | --- |
| *VfSAD1* | TGGGGTCTATACTGCGAAGG | ACTGAAAGGGACGATGGTTG | 196 bp |
| *VfSAD2* | GTGCGAGCCTTACTTCTTGG | TTTTCGGATCCATTCCTGAG | 165 bp |
| *AtWRI1* | ACGTACGATCTGGCTGCTCT | CATCTTCCGTTGTGGTGATG | 203 bp |
| *AtDGAT1* | TTTGGCCGGAGATAATAACG | CGTCGGAGCTAAGTGGACTC | 149 bp |
| *AtACT2* | GCCATCCAAGCTGTTCTCTC | CAGTAAGGTCACGTCCAGCA | 157 bp |

**Table S2. Analysis of the physicochemical properties of** **VfSAD1and VfSAD2 proteins.**

| Protein name | *VfSAD1* | *VfSAD2* |
| --- | --- | --- |
| Full length of the sequence | 1191 bp | 1176 bp |
| Protein length (aa) | 397 | 386 |
| Molecular formula | C_3601_H_6013_N_1191_O_1507_S_247_ | C_3541_H_5908_N_1176_O_1477_S_255_ |
| Total number of atoms | 12559 | 12357 |
| Relative molecular weight (KDa) | 45.54 | 43.82 |
| Theoretical isoelectric point | 5.05 | 5.04 |
| Stability | Instability | Steadility |

**Fig.S1. Schematic diagram of fatty acid biosynthesis in seeds of Tung tree.**


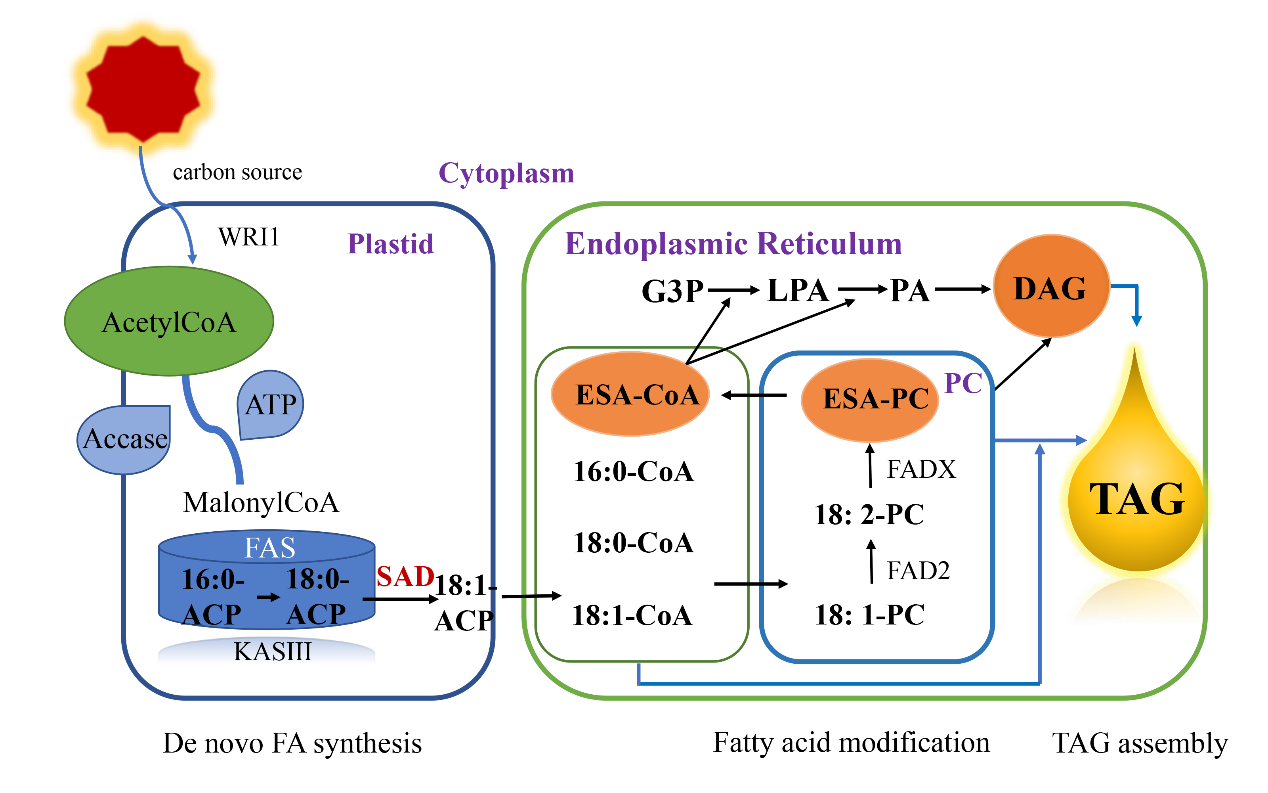


**Fig.S1**. Schematic diagram of fatty acid biosynthesis in seeds of Tung tree.

The Tung tree is like any other oil plant, the carbon source is introduced into the plant cytoplasm and provides the final source of carbohydrates for conversion to acetyl-CoA. KASIII initiates FAs synthesis by condensation of acetyl-CoA and malonyl-ACP, KASI continues elongation to C16:0, and KASII provides the final elongation step from C16:0 to C18:0. 18:0 ACP is desaturated by a stearoyl-ACP desaturase (SAD) to generate 18:1 ACP. After a series of reactions, acyl chains are transferred to ER for glycerin assembly. Finally, the TAG is synthesized in the endoplasmic reticulum and assembled in the oil body.

(ACCase, acetyl CoA carboxylase; The plant FA synthase (FAS), fatty acid synthase; SAD, stearoyl-ACP desaturase; FA, fatty acid; ACP, acyl carrier protein; PC, phosphatidylcholine; FAD, fatty-acid desaturase; TAG, triacylglycerol); FAD2, Oleate desaturase; G3P, glycerol-3-phosphate; PA, phosphatidic acid.

**Fig. S2. The characteristic analysis of VfSAD1 and VfSAD2 protein.**

**
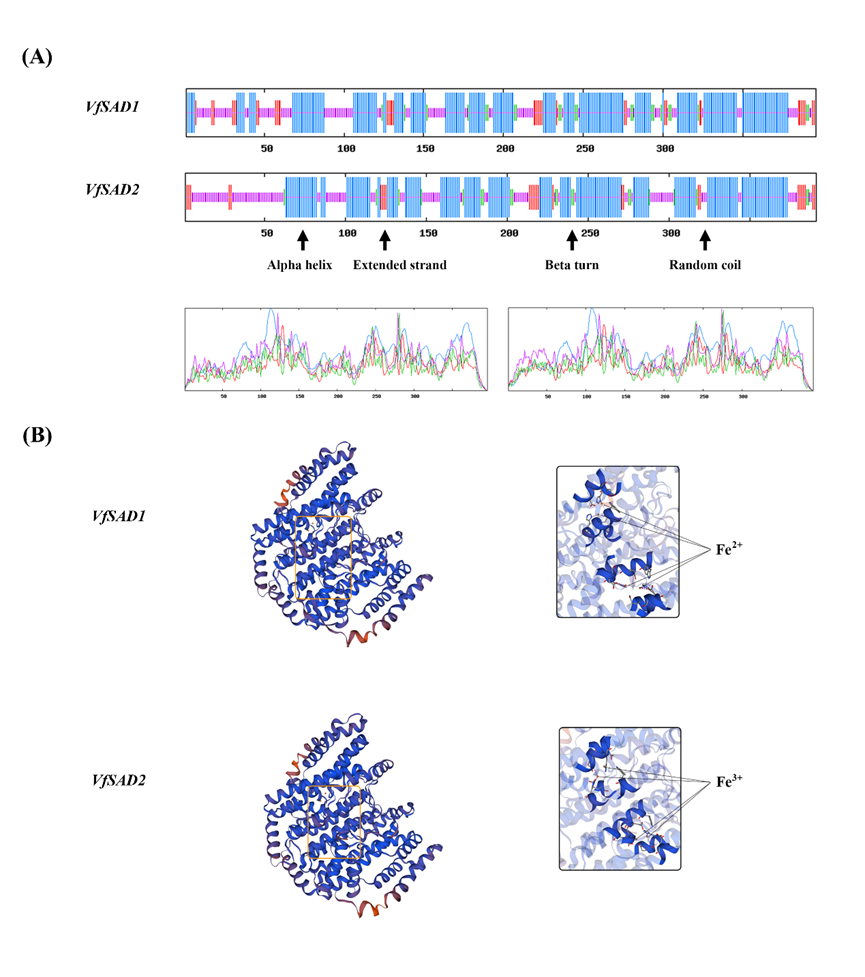
Fig. S2.** The characteristic analysis of VfSAD1 and VfSAD2 protein. The secondary structure **(A)** and tertiary Structure **(B)** of VfSAD1 and VfSAD2. The arrows point to the way in which the protein polypeptide chains themselves fold and coil. The box is the two irons center of the VfSAD1 dimer and the three iron center of the VfSAD2 dimer, and the ligand of the Fe atom is the amino acid side chain or group of the four-helix bundle.

**Fig. S3. Multiple sequence alignment and phylogenetic tree analysis of SAD proteins.**


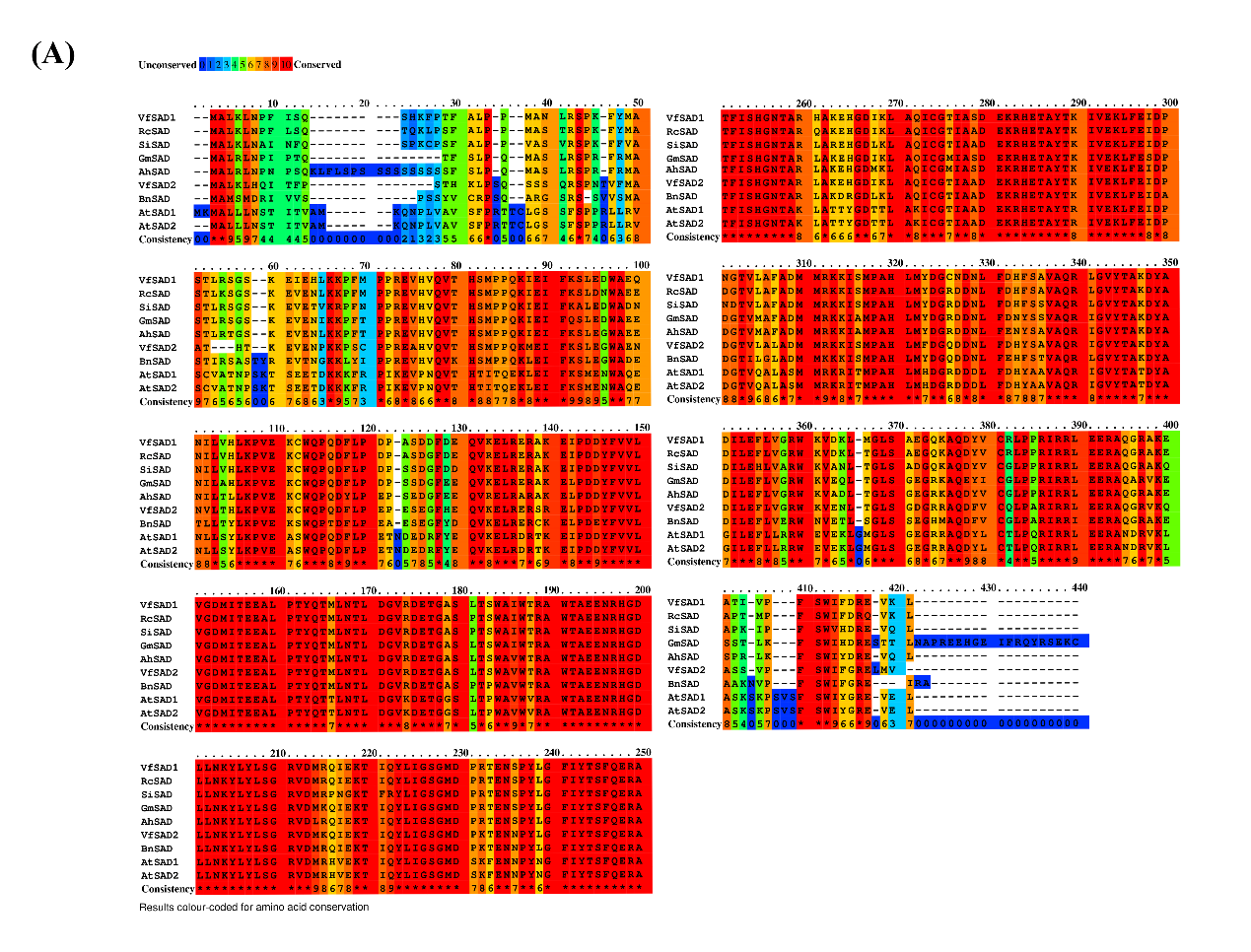


**Fig. S3.** Multiple sequence alignment of SAD proteins. **(A)** Multiple sequence alignment of the amino acid sequences of SAD proteins from Tung tree and other species.
